# Rezum water vapor thermal therapy for treatment of lower urinary tract symptoms: A retrospective single-centre analysis from a German high-volume centre

**DOI:** 10.1371/journal.pone.0279883

**Published:** 2023-01-06

**Authors:** Thorben Winkler, Christoph A. J. von Klot, Stephan Madersbacher, Markus A. Kuczyk, Mathias Wolters

**Affiliations:** 1 Department of Urology and Urologic Oncology, Hannover Medical School (MHH), Hannover, Germany; 2 Department of Urology, Klinik Favoriten, Vienna, Austria and Department of Urology, Sigmund Freud Private University, Vienna, Austria; Weill Cornell Medical College, UNITED STATES

## Abstract

**Objective:**

Rezum is a minimal invasive surgical treatment for patients with lower urinary tract symptoms (LUTS) related to benign prostatic enlargement (BPE). The aim of our study was to assess safety and efficacy of the Rezum procedure in a consecutive series of patients.

**Material and methods:**

A retrospective study was performed in a single tertiary care hospital in patients undergoing Rezum procedure between 2018 and 2020. All patients that underwent intervention because of drug-refractory moderate to severe LUTS were assessed. Descriptive outcomes such as symptoms scores (IPSS, IPSS-QoL), peak flow in uroflowmetry (Qmax), post-micturition residual urine volume (PVR) and prostate volume (PVol) were analysed.

**Results:**

In total, 92 Rezum procedures were performed in the observational period. All interventions were competed without device- or procedure relates adverse events. Patients achieved a significant symptom relief as measured in IPSS (50% reduction, p<0.001, n = 35) and IPSS-QoL score (53% reduction, p<0.001, n = 35). Qmax improved by 7.3 ml/s from 10.6 ± 4.2 ml/s to 17.9 ± 9.3 ml/s (p = 0.003, n = 20) were as mean PVR significantly decreased by 136 ml from 175 ± 194.1 to 39 ± 62 ml (p = 0.007, n = 20). PVol significantly decreased by 40.3% from 73.9 ± 41.2 to 44.9 ± 29 ccm (p = 0.024, n = 17). All pre-interventional catheter-depending patients (28.3% of all patient) were catheter independent after six weeks.

**Conclusion:**

Rezum therapy is safe and effective and can be considered a viable treatment option for BPH related LUTS.

## Introduction

Benign prostatic hyperplasia (BPH)/benign prostatic enlargement (BPE) is a chronic disorder that is often associated with the development of lower urinary tract symptoms (LUTS). Its prevalence is rising worldwide as average life expectancy increases [1]. Most common LUTS are based on bladder outlet obstruction (BOO) as a result of BPE affecting health-related quality of life [2]. Early-stage treatment consists of lifestyle modifications as well as medication. After failure of medical therapy or treatment break off due to side effects, surgical treatment can be offered. Since decades transurethral resection of prostate (TURP) is the standard of care for moderate to serve drug-refractory LUTS in patient with prostate volumes up to 80 ccm. TURP is associated with perioperative morbidity rates (up to 20%) and frequent postoperative complications (i.e. urethral stricture up to 7% and incontinence up to 3%) [3].

Over the last decades serval treatment alternatives for LUTS due to BPE/BPO with a broad range of invasiveness and efficiency were introduced [4]. Radiofrequency-generated water vapour thermal therapy, marketed as Rezum System, distributed by Boston Scientific (Marlborough, MA, US), is one of the latest minimal invasive options for BPH treatment [5,6]. Rezum system has been approved by the United States Food and Drug Administration (US FDA) in 2015 (510(k) number: K150786). Convective water vapor thermal energy is generated via radiofrequency and injected with a retractable needle in the prostatic tissue under cystoscopic control. Rapid change in tissue temperature causes immediate cell necrosis in treated areas of the prostate tissue. Maximum effect is expected after an interval of 6 weeks to 3 months postoperatively. The most relevant postulated advantages of this technique are the short operation time, the superior safety profile and the good preservation of sexual function. In contrast to some other minimal invasive therapies (e.g. Urolift) Rezum procedure can be applied in patients with a median prostate lobe [7]. AUA stated that Rezum therapy may be offered to patients with LUTS attributed to BPH provided prostate volume <80g [8]. Other national organisations e.g. British National Institute for Health and Care Excellence (NICE) mentioned the therapy in similar ways whereas EAU did not mention Rezum therapy yet.

Recently data form the largest clinical trial yet reported significant improvements in patient-reported symptom relief and quality of life as well as uroflowmetry measurements for 5-year follow up [9].

In our study we aimed to evaluate the clinical outcome of the first cohort of patients treated with the Rezum system at our university hospital to provide more evidence on the efficiency of the method.

## Material and methods

### Study population

Study population consisted of 92 consecutive patients with symptomatic BPH that were treated with the Rezum system in the Department of Urology and Urologic Oncology at Hannover Medical School, Germany. Data collection was performed from 2018 to 2020. Inclusion criteria were age >40 years as well as moderate to severe drug-refractory LUTS defined by International Prostate Symptom Score (IPSS) (8–19 points and 20–35 points). Exclusion criteria were prostate cancer and any active urinary tract infection as well as any neurogenic bladder disorders according to current diagnosis guidelines. Patients’ characteristics at baseline are shown in Table 1. All patient related data were fully anonymized and analyzed retrospectively in full compliance with the regulations for retrospective studies set up by the local institutional review board (study number 10234_BO_K_2022) and in accordance with the Declaration of Helsinki. All study participants gave their written, informed consent in the analysis of clinical data for medical research. Patients’ characteristics are summarized in Table 1.

**Table 1:** Patients’ characteristics at baseline and interventional data. Values are mean ± standard deviation (SD). BCD = bladder neck-colliculus-distance (cm). Min = minimum. Max = maximum.

	Mean ± SD	Min	Max
Age (years)	70.88 ± 9.82	42	93
Prostate volume (ccm)	65.42 ± 32.51	20	166
BCD (cm)	4.6 ± 1.44	2.5	9
Rezum procedures left prostate lobe	4.37 ± 1.5	2	9
Rezum procedures right prostate lobe	4.14 ± 1.43	2	8
Rezum procedures median prostate lobe/central zone (n = 51)	2.59 ± 1.17	1	6

### Study protocol and clinical assessment

The following clinical data have been assessed for all patients prior to treatment. Follow up assessments were offered on a voluntary basis three and six months after treatment.

IPSS and IPSS-Quality of Life (QoL) score were assessed by standardised questionnaires. Patients with transurethral indwelling catheter before treatment were asked to complete scores as they assess before catheter maintenance. Uroflowmetry was performed in standardised way by qualified and trained urology nurses. Post-micturition residual urine volume (PVR) was evaluated by transabdominal sonography of the bladder. Prostate volume (PVol) was measured by transrectal sonography of the prostate (TRUS). All sonography examinations were performed by an experienced physician unaware of the study protocol and were documented in medical records. Device- or procedure relates adverse events were assessed using the Clavien-Dindo classification system [10].

### Operative procedure

The Rezum system for water vapour thermal therapy was used following manufacturers recommendations for treatment of both prostatic lobes as well as central zone or median lobe as previously reported [11]. Interventional data are shown in Table 1. All interventions were performed under general anaesthesia. Post-interventional all patients received a transurethral indwelling catheter which was removed before discharge of hospital. Pre-interventional catheter-dependent patients either received a suprapubic catheter or maintained with transurethral catheter until sufficient voiding was assured post-interventional.

### Statistical analysis

Statistical analysis was carried out in R. For comparison of data before and after treatment a Two-Sample t­test was used. Pre-interventional data were obtained up to 200 days before intervention. Post-interventional data were obtained within the range of 50 to 300 days after intervention. In case more than one data existed, subsequent post-interventional data were selected for analysis.

## Results

In total, 92 patients were treated in the observational period. Mean patients age was 70.9 ± 9.8 years (mean ± SD), mean prostate size at baseline was 65.4 ± 32.5 ccm. 26 patients (28.3%) were catheter-dependent pre-interventional. Mean follow up was 137 ± 55 days. All results are summarized in Table 2.

**Table 2:** Patients’ characteristics before and after treatment. Values are mean ± standard deviation (SD). Mean follow-up: 137 ± 55 days. IPSS = International Prostate Symptom Score. QoL = IPSS Quality of Life Score. TRUS = transrectal sonography of the prostate.

	Before Therapy	After Therapy	P-Value
IPSS Score (n = 35)	19.34 ± 6.06	9.74 ± 6.41	<0.001
QoL Score (n = 35)	4.14 ± 1.17	1.94 ± 1.19	<0.001
Maximum flow in uroflowmetry, Qmax (ml/s) (n = 20)	10.57 ± 4.15	17.9 ± 9.33	0.003
Prostate volume in TRUS (ccm) (n = 17)	73.94 ± 41.17	44.88 ± 29.03	0.024
Post-micturition residual urine, PVR (ml) (n = 20)	175 ± 194.06	39 ± 61.97	0.007

All interventions were competed without device- or procedure relates adverse events. Study patient received on an average 8–9 water vapour injection (range 2 to 18). 51 patients (55%) received treatment of a median prostate lobe. Mean operative time was 8:54 minutes. Mean length of hospital stay was 3.43 ± 1.73 days. 71% of patients (n = 66) were discharged from hospital without urinary catheter. Nine patients (9.8%) were discharged with transurethral catheter and 17 patients (18.5%) were discharged with a suprapubic catheter. All urinary catheters could be removed in an outpatient setting within the first six weeks after treatment.

**Fig 1:**
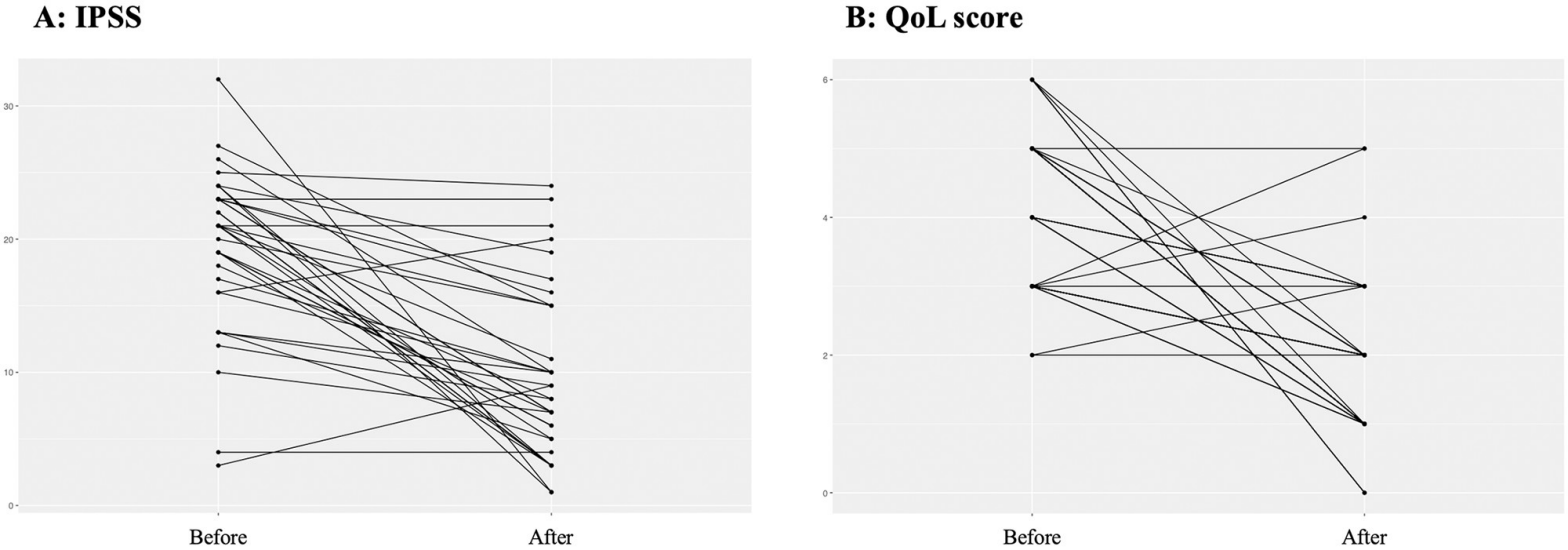
(A) International Prostate Symptom Score (IPSS) before and after treatment (p<0.001, n = 35). (B) Quality of Life (QoL) score before and after treatment (p<0.001, n = 35).

Regarding overall patient-reported outcomes, LUTS improved significantly as measured in IPSS and QoL score. IPSS improved by 50% from baseline 19.3 ± 6.1 to 9.7 ± 6.4 points (p<0.001, n = 35; Fig 1A). In line QoL score improved by 53% from baseline 4.1 ± 1.2 to 1.9 ± 1.2 points (p<0.001, n = 35; Fig 1B).

**Fig 2:**
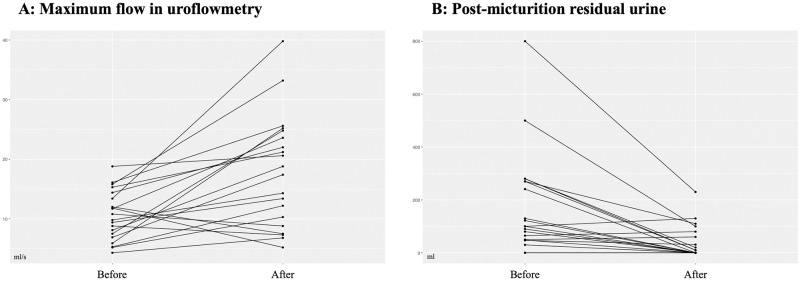
(A) Maximum flow (ml/s) in uroflowmetry before and after treatment (p = 0.003, n = 20). (B) Post-micturition residual urine (ml) before and after treatment (p = 0.007, n = 20).

Mean Qmax significantly improved from 10.6 ± 4.2 ml/s to 17.9 ± 9.3 ml/s (p = 0.003, n = 20, 41% improvement; Fig 2A) post-interventional were as PVR significantly decreased from 175 ± 194 ml to 39 ± 62 ml (p = 0.007, n = 20; Fig 2B).

**Fig 3:**
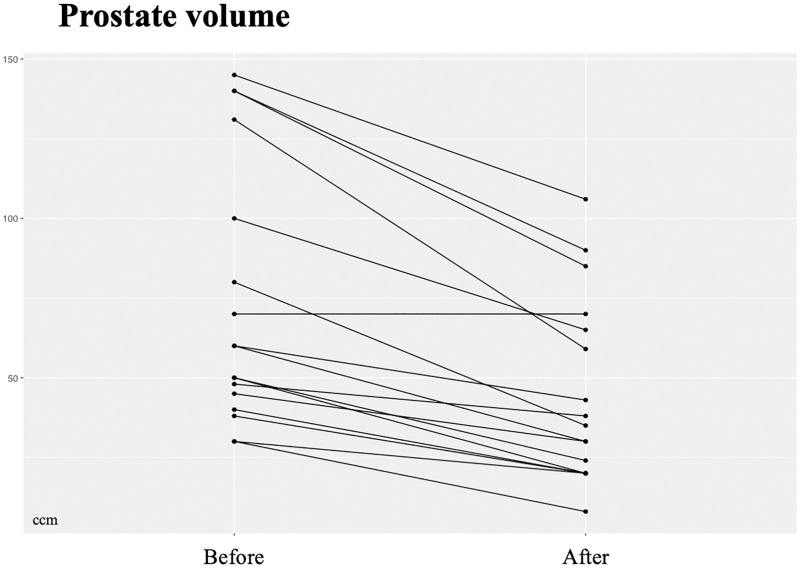
Prostate volume (ccm) before and after treatment (p = 0.024, n = 17).

Prostate volume in TRUS significantly decreased from 73.9 ± 41.2 ccm to 44.9 ± 29 ccm in (p = 0.024, n = 17; Fig 3).

Focusing on the subgroup of pre-interventional catheter-dependent patient, only improvements in QoL were statistically significant (4.6 to 1.3, p<0.001, n = 7). A subgroup of patient with pre-interventional large prostate sizes <80 ccm likewise showed improvements in QoL (3.9 to 2, p = 0.003, n = 9) as well as significant decrease in PVol by 40% (122.7 to 73.4 ccm, p = 0.008, n = 6).

## Discussion

We aimed to assess the effectiveness of water vapour thermal therapy of the prostate, marketed as Rezum system, in a consecutive series of patients. Our cohort represents one of the largest single centre series. Overall, Rezum therapy seems to be a promising option in LUTS treatment on a minimal invasive approach.

In our study, we could confirm effectiveness and safety in line with a previously published prospective randomised control trail [12], some retrospective series [13–16] as well as a prospective pilot study [17].

Patient-reported outcome as reflected in IPSS and IPSS-QoL score improvements by approx. 50% were corresponding to observations by McVary et al. (46.7% reduction in IPSS and 42.9% reduction in IPSS-QoL) or Darson et al. (54.2% reduction in IPSS).

Reflecting changes in uroflowmetry our findings outline an enhancement in maximum urinary flow (Qmax) by nearly 41%, which seems appropriate as compared to 49.5% improvement according to McVary et al. and 51.4% improvement reported by Darson et al. Concerning post-micturition residual urine volume (PVR) in non-retention patients an 78% reduction as shown seems to be a considerable effect in comparison to recently published data (PVR reduction: McVary et al.: 38%, Mollengarden et al.: 32.3%, Darson et al.: 34.9%).

Of note, we could outline in our data that prostate volume as measured by transrectal sonography of the prostate decreases by more than one third in the first quarter year after therapy. Most of the data reflecting the outcomes of Rezum therapy did not capture effects on prostate volume. A trail published by Mollengarden et al. reported a reduction of prostate volume by 17% after Rezum therapy. Nevertheless, effect on prostate volume is probably linked to numbers of treatment procedures per intervention. In this study patient received on an average 8–9 water vapour injection (range 2 to 18).

Notably, we treated 18 patients (19.6%) with large prostate volumes >80 ccm. Our analysis indicates that Rezum treatment seems to induce similar improvements in symptom score and flow rates as reported in small prostates <80 ccm. This provides further evidence to a recently published study verifying Rezum therapy effects regardless of prostate size [13].

In our study, we could show that 26 pre-interventional catheter-dependent patients (28% of all patients) were catheter free after six weeks. In line with recently published data reporting a catheter free rate of 70% at a median of 26 days [18] resp. 93% after 3 months [19], we could indicate that Rezum therapy can be taken into account for these patients.

Based on our clinical experience, Rezum seems to be a good treatment option especially for young patients with bothersome symptoms and/or failure/side effects of medical BPH treatment as well as older patients with numerous comorbidities that have considerable advantages from short operative times.

### Limitations of this study

Major limitations of this study are the retrospective design as well as the number patients lost to structural follow up in our clinic largely due to structural division of in- and outpatient healthcare system in Germany. Likewise, the overall small sample size, single centre design as well as limited follow up have to mentioned as limitations of the study. Nevertheless, further prospective largescale studies are necessary to prove our findings.

## Conclusion

Our single-centre retrospective analysis confirms that Rezum is an effective and safe minimal invasive therapeutic option for patients with BPH related LUTS. It is a promising novel technique that can also be performed in patients with median lobe.
